# Bilateral Transcranial Direct Current Stimulation Reshapes Resting-State Brain Networks: A Magnetoencephalography Assessment

**DOI:** 10.1155/2018/2782804

**Published:** 2018-01-11

**Authors:** Giovanni Pellegrino, Matteo Maran, Cristina Turco, Luca Weis, Giovanni Di Pino, Francesco Piccione, Giorgio Arcara

**Affiliations:** ^1^San Camillo Hospital IRCCS, Venice, Italy; ^2^NeXT: Neurophysiology and Neuroengineering of Human-Technology Interaction Research Unit, Campus Bio-Medico University, Rome, Italy

## Abstract

Transcranial direct current stimulation (tDCS) can noninvasively induce brain plasticity, and it is potentially useful to treat patients affected by neurological conditions. However, little is known about tDCS effects on resting-state brain networks, which are largely involved in brain physiological functions and in diseases. In this randomized, sham-controlled, double-blind study on healthy subjects, we have assessed the effect of bilateral tDCS applied over the sensorimotor cortices on brain and network activity using a whole-head magnetoencephalography system. Bilateral tDCS, with the cathode (−) centered over C4 and the anode (+) centered over C3, reshapes brain networks in a nonfocal fashion. Compared to sham stimulation, tDCS reduces left frontal alpha, beta, and gamma power and increases global connectivity, especially in delta, alpha, beta, and gamma frequencies. The increase of connectivity is consistent across bands and widespread. These results shed new light on the effects of tDCS and may be of help in personalizing treatments in neurological disorders.

## 1. Introduction

Transcranial direct current stimulation (tDCS) is a noninvasive neurostimulation technique capable of modulating brain excitability and inducing plastic phenomena outlasting the duration of the stimulation itself [1–3].

tDCS consists in the application of a weak homogeneous direct current over the scalp using two electrodes of different polarity (anode and cathode) connected to a stimulator, decreasing the cortical excitability under the cathode and increasing it under the anode [4]. Because of its ease of use, limited side effects, and low cost [5], tDCS has become very popular in the recent years and has been applied in a number of different frameworks, ranging from cognitive and social neuroscience [6] to clinical research [7]. tDCS application is now explored as a promising tool for the treatment of drug-resistant epilepsy [8] and, together with physical therapy, to boost brain plasticity and possibly to improve the outcome of disabled stroke patients [9–11].

As it happens for other noninvasive brain stimulation techniques, the mechanism by which tDCS is supposed to work is mainly related to the long-lasting changes of brain excitability [2, 12, 13]. However, change of excitability is disclosing only one aspect of tDCS effects, which surely involve modulation of neurotransmission [14], of brain activity [15], and of metabolism [16–18].

Despite the efficacy of both noninvasive and invasive brain stimulations in treating multiple neurological and psychiatric conditions being strictly dependent on their effects on resting-state brain networks [19], very little is known so far on tDCS effects on brain activity and connectivity.

In this study, we focused on the effects of tDCS on resting-state brain networks as assessed by magnetoencephalography (MEG). MEG is a noninvasive technique measuring cortical magnetic activity with high temporal and spatial resolution [20–24]. Compared to other techniques such as EEG, MEG also owns the unique advantage of detecting signals without the application of electrodes on the scalp, thus allowing to place and to activate/deactivate tDCS without significant interference with the acquisition process.

We designed a sham-controlled, double-blind study where healthy subjects were scanned immediately before and after a 20-minute session of bihemispheric tDCS to investigate the effects of tDCS on the architecture of brain networks. Since both cathode and anode are active in producing cortical effects, the bihemispheric montage tested in this study exploits mechanisms of interhemispheric interaction to enhance the biological effects of tDCS [8, 9].

## 2. Materials and Methods

### 2.1. Participants and Experimental Design

**Figure 1:**
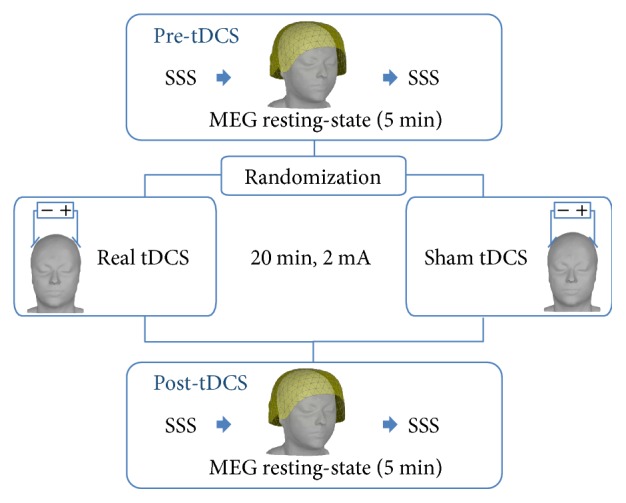
Experimental design.

We recruited 15 healthy subjects (mean age = 28.8 ± 3 (2 SE); 12 F) to participate in a randomized, sham-controlled, double-blind tDCS study (Figure 1). Each participant underwent two sessions of bihemispheric tDCS stimulation (sham and real). The two sessions were at least 20 h far apart. Before and after each tDCS session, we measured resting-state MEG data for about 5 minutes. All the subjects were right-handed as assessed by the Oldfield's Edinburgh inventory (91.13 ± 6.8) [25] and were free from medications. The fluctuations of vigilance were controlled by means of the Stanford Sleepiness Scale (SSS), which was administered before and after every MEG scan [26]. The experimental procedures were carried out at the MEG unit of the IRCCS San Camillo hospital in Venice, with the subjects lying down on a bed in a supine position in a quiet environment. Subjects were asked to keep their regular wake/sleep cycle before participation. All the procedures were performed in agreement with the 1964 Helsinki Declaration and its later amendments. This study was approved by the local ethics committee, and all participants provided a written informed consent.

### 2.2. tDCS

tDCS was delivered with a battery-powered stimulator connected to a pair of saline-soaked sponge electrodes having a surface of 35 cm^2^. Real/sham stimulation was applied over the sensorimotor regions bilaterally, with the cathode (−) centered over C4 and the anode (+) centered over C3, where C3 and C4 are scalp positions according to the 10/20 international EEG system. This montage has been previously employed for clinical applications [8, 9]. Real stimulation lasted 20 minutes with 20 seconds of fade-in and fade-out, an intensity of 2 mA, and the current density was 0.057 mA/cm^2^. For the sham stimulation, we employed the same setting except for the current, which was only applied for 20 seconds at the beginning and at the end of the stimulation with the aim of giving a slight tingling sensation that many subjects report for tDCS real stimulation.

### 2.3. MEG Data Acquisition and Preprocessing

MEG measures were acquired with a CTF MEG system (MISL, Vancouver, Canada) with 275 MEG gradiometers. Eye blinks, eye movements, and electrocardiogram (EKG) were recorded using bipolar electrodes, and the head position within the helmet was continuously monitored thanks to three localization coils placed on anatomical landmarks (the nasion and the left and right ear canals). The sampling rate was set to 1200 Hz. The acquisition lasted 5 minutes. Subjects were scanned with their eyes closed and were given the following instructions: “Clear your mind and stay relaxed.” Before and after each MEG acquisition, the technician administered the Stanford Sleepiness Scale [26]. MEG data analysis was performed with Brainstorm toolbox [27], which is documented and freely available for download online under the GNU general public license (http://neuroimage.usc.edu/brainstorm). The preprocessing pipeline consisted of (1) third-order spatial gradient noise cancellation, (2) downsampling to 600 Hz, (3) signal space-separation (SSP), (4) epoching, (5) DC removal, and (6) bad sensor removal [22]. Artifacts related to heartbeat and eye movements were removed in step 3 of the pipeline using the SSP procedure [28, 29]. Resting-state signals were divided in step 4 into 20-second-lasting epochs. Each epoch was visually inspected, and those affected by artifacts were rejected.

### 2.4. Source Imaging

For each participant, we acquired an individual whole-head 3-dimensional sagittal T1-weighted-3D-TFE scan with a 1.5 T Achieva Philips scanner (Philips Medical Systems, best, Netherlands), with the following scan parameters: repetition time (TR) = 8.3 milliseconds, echo time (TE) = 4.1 milliseconds, flip angle = 8°, acquired matrix resolution (MR) = 288 × 288, and slice thickness (ST) = 0.87 mm. The cortical mesh of the “mid” cortical layer equidistant from white/grey matter interface and pial surface was segmented using FreeSurfer software [30], tasselled into 15,000 vertices, and then downsampled to 8000 vertices, whereas the reconstruction of the skull surface and the coregistration between patients' MRI and MEG data was performed with the Brainstorm toolbox [27]. The individual head model for source imaging was implemented with the OpenMEEG boundary element method (BEM) [31]. We only considered one cortical layer with a conductivity of 0.33 S/m. The inverse problem was solved by using a whitened and depth-weighted linear L2-minimum norm estimate algorithm, with the estimated dipole orientations constrained to be normal to the cortex. A common imaging kernel was computed and then applied to obtain single epoch cortical reconstructions. Noise covariance for source reconstruction was obtained from an empty room recording of 2 minutes.

### 2.5. Brain Network Analysis: Resting-State Activity and Connectivity

To assess the changes in brain networks, we focused on two aspects: resting-state activity and connectivity.

Firstly, to have a general measure of resting-state activity, we focused on the spectral power of specific bands. Specifically, we calculated power spectrum density (PSD) at the source level. After inverting the signal onto the cortical surface, we computed the PSD for each cortical vertex in all the relevant frequency bands (delta: 2–4 Hz; theta: 5–7 Hz; alpha: 8–12 Hz; beta: 15–29 Hz; and gamma: 30–60 Hz).

We also focused on measuring resting-state connectivity across the brain, estimating the changes in coupling between two seeds beneath the tDCS electrodes and the rest of the cortex. We computed the phase locking value (PLV) [32], which is a very popular measure of brain synchronization commonly used to estimate nondirectional functional connectivity [33]. The connectivity analysis was performed considering two cortical seeds, underneath the cathode and the anode. They corresponded to the left and right primary sensorimotor hand regions. These regions were manually drawn by an expert neurologist (GP) onto the individual cortical surface using anatomical landmarks [34]. Each seed was extended about 10 cm^2^. The signal within each seed was averaged, and the connectivity between such an average and every other cortical vertex was computed before and after tDCS. The same procedure was performed for the following frequency bands (delta: 2–4 Hz; theta: 5–7 Hz, alpha: 8–12 Hz; beta: 15–29 Hz, gamma: 30–60 Hz). To allow group analysis, PSD and PLV maps were projected onto standard MNI template [35] and spatially smoothed with full width at half maximum at 3 mm [36], which is the default value in Brainstorm for MEG and is compatible with the image resolution and distribution provided by the minimum norm estimate.

### 2.6. Statistical Analysis

Statistical analysis was performed using the IBM SPSS Statistics (ver. 24) and Matlab (Mathworks). After checking data distribution using the Kolmogorov and Smirnov test, Stanford Sleepiness Scale scores were modeled using a repeated measure ANOVA, with factor time (4 levels) and stimulation (2 levels) (Figure 1). For activity and connectivity analysis, after checking that the baseline (pre-tDCS) measures were not different between stimulations, the post-tDCS measures were expressed as percentage of the pre-tDCS according to the following formula (post-tDCS−pre-tDCS)/pre-tDCS∗100. Post-tDCS variations of activity and connectivity were directly compared between the real and sham stimulations by means of *t*-statistics. This procedure was applied for each vertex of the cortical surface, and it allowed to generate *t*-maps of real minus sham differences. In order to test whether tDCS was inducing a global increase or decrease of activity/connectivity, we applied a Wilcoxon test comparing the actual *t*-value distribution with a theoretical distribution with 0 mean and same variability as the one empirically found. In other words, we tested whether the distribution was significantly different from the one expected under the null hypothesis of no global tDCS effect. Then, we visually explored the spatial distribution of the effects. As further exploratory analysis of the topographic effects, we extracted average measures of PSD and PLV from regions of interest (ROIs) derived from a parcellation of the cortical surface implemented in Brainstorm [37] and we compared the real versus sham stimulations by means of *t*-tests. These results are reported in the Supplementary Materials (available here).

## 3. Results

All participants completed the experimental sessions, and none of them reported any problem or discomfort during the tDCS procedure or during the MEG recordings. Moreover, none of the participants reported to have clearly identified the real or sham session.

As for the sleepiness evaluation, the repeated measure ANOVA showed no significant factor stimulation nor time by stimulation interaction (*p* > 0.200 consistently). We did find a significant factor time [*F* (3,42) = 6.596, *p* = 0.001]. This effect was related to a sleepiness increase between the beginning and the end of the MEG resting-state scan. The size of the effect was small (about 1 point) and the average values at all time-points were always below 2.5, suggesting that subjects were awake during the entire study.

### 3.1. Resting-State Brain Activity (PSD)

**Figure 2:**
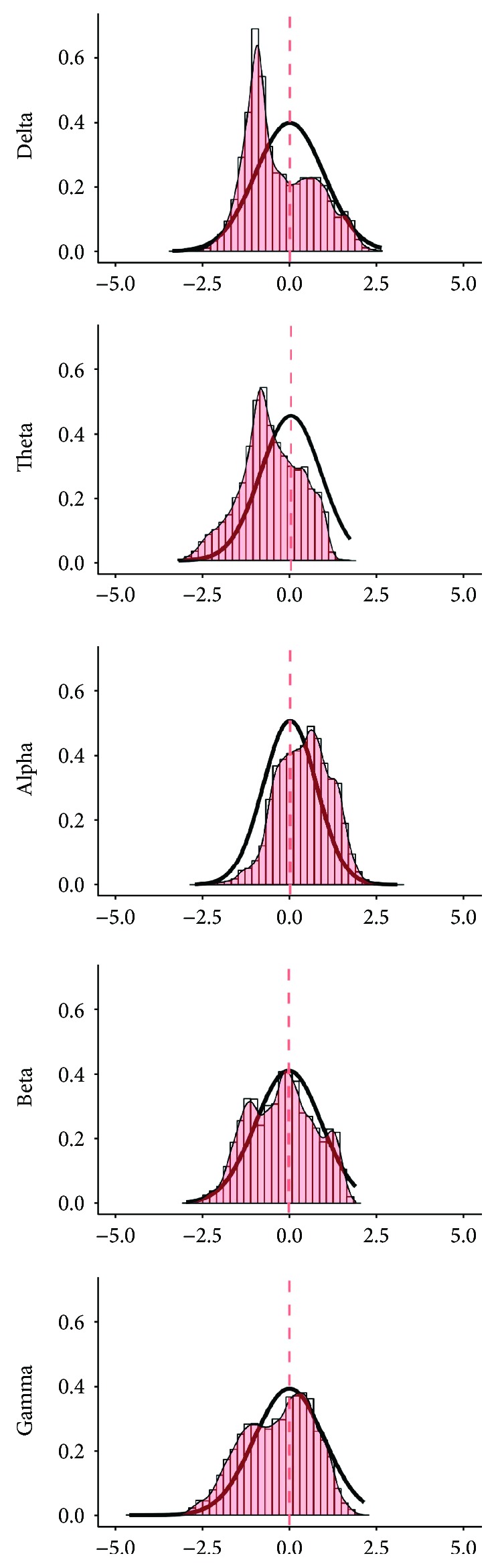
Resting-state activity (PSD) cortical *t*-value distribution. Histograms with red bars and the superimposed density plot with the thin line were calculated from the observed *t*-value distribution for the frequency bands under investigation. The tick black line shows a theoretical null-hypothesis distribution, with zero mean and zero median and the same variability of the empirical distribution. *x*-axes: *t*-value; *y*-axes: frequency of cortical vertices exhibiting a specific *t*-value expressed as proportion on the entire sample (intensity).

**Figure 3:**
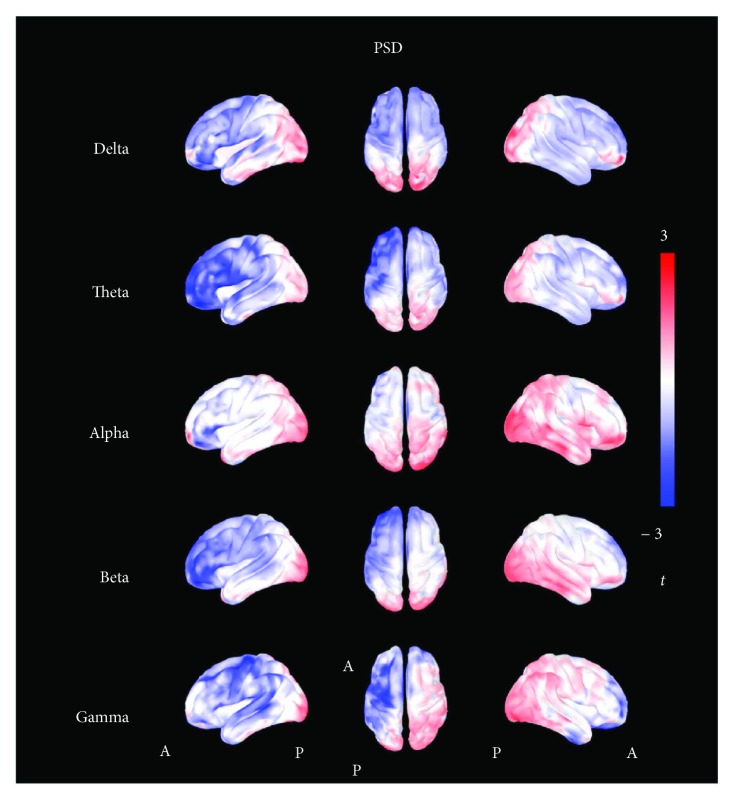
*t*-maps of resting-state activity (PSD). The figure shows the topographic distribution of *t*-values calculated for the power spectrum density (PSD). Shades of red colors indicate positive values, shades of blue colors indicate negative values, and shades of white indicate values toward zero. The *t*-maps show a global reduction of PSD in all frequency bands, mostly localized in the left frontal regions.

The results of the analysis on brain activity (i.e., PSD) are also reported in Figures 2 and 3. The analysis showed that all *t*-maps had a distribution significantly different from the theoretical null distribution [delta U = 36.111^∗^10^6^; theta U = 19.312∗10^6^; alpha U = 86.411∗10^6^; beta U = 44.582∗10^6^; gamma U = 38.501∗10^6^, *p* < 0.001 consistently]. The empirical *t*-value distributions were mostly shifted to negative values for the delta, theta, beta, and gamma frequency bands, and only slightly positively shifted for the alpha band (Figure 2). The effect was stronger for delta, theta and alpha band and, even if it was statistically significant, less evident for beta and gamma bands, whose curves of empiric distribution were very close to those theoretically generated.

The evaluation of the topographic distribution indicated a stronger effect, consistent across multiple frequency bands, in the left frontal regions, homolateral to the anode (+) (Figure 3). The ROI-based analysis confirmed such finding. The reader is referred to the supplementary materials for the ROI-based analysis of the spatial distribution of the effects.

### 3.2. Resting-State Brain Connectivity (PLV)

**Figure 4:**
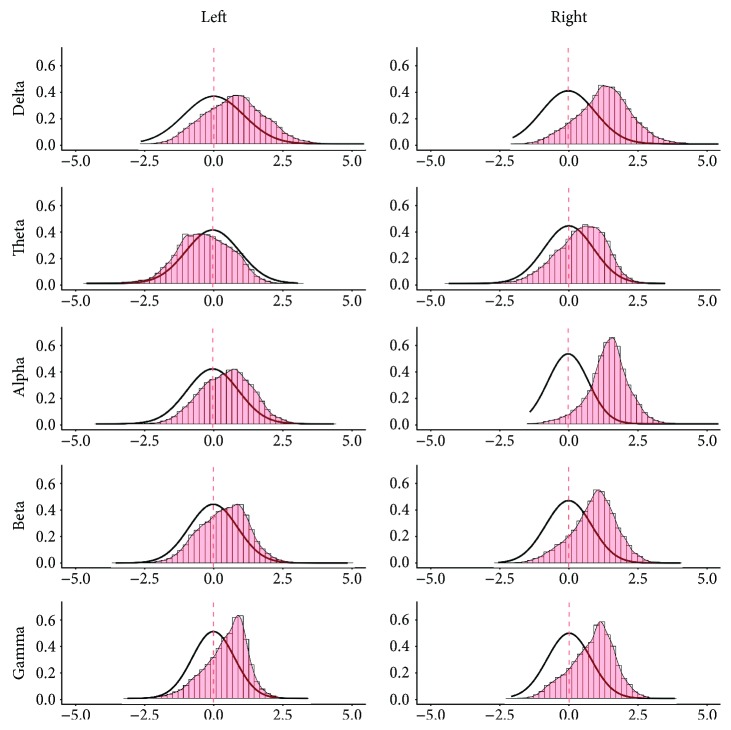
Resting-state connectivity (PLV) cortical *t*-value distribution. The figure shows the distribution of the *t*-values for the phase locking value maps calculated at the source level. The histogram with red bars and density plot with the thin line were calculated from the observed *t*-value distribution. The tick black line shows the theoretical null-hypothesis distribution, with zero mean and the same variability of the empirical distribution. Results are presented in an array divided in rows and columns (bands by seeds). *x*-axes: *t*-value; *y*-axes: frequency of cortical vertices exhibiting a specific *t*-value expressed as proportion on the entire sample (intensity).

**Figure 5:**
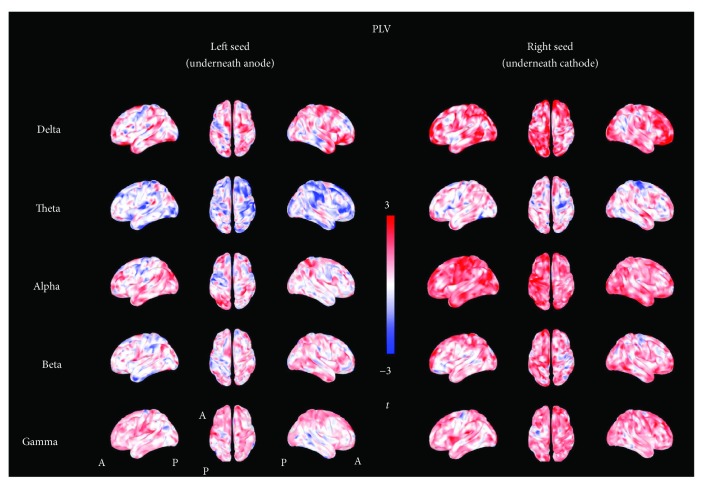
*t*-maps of resting-state connectivity (PLV). The figure shows the topographic distribution of *t*-values calculated for the phase locking value. Shades of red colors indicate positive values, shades of blue colors indicate negative values, and shades of white indicate values toward zero. An inspection of these maps shows a widespread increment of PLV values in all frequency bands.

The results of the analysis on brain connectivity (i.e., PLV) are also reported in Figures 4 and 5. The analysis showed that all *t*-maps had a distribution significantly different from the theoretical null distribution for both the left seed (under the anode) and right seed (under the cathode) [delta: left *U* = 93.770^∗^10^5^, right *U* = 109.13∗10^6^; theta: left *U* = 38.04∗10^6^, right *U* = 85.342∗10^6^; alpha: left *U* = 87.201∗10^6^, right *U* = 111.94 ∗10^6^; beta: left *U* = 83.063∗10^6^, right *U* = 10.489∗10^6^; gamma: left *U* = 90.813∗10^6^, right *U* = 10.414∗10^6^, *p* < 0.001 consistently].  Figure 4 shows that the observed *t*-value distributions were shifted toward a positive effect, which indicates overall higher connectivity values after real stimulation as compared to sham. In particular, the distribution was positively shifted for both seeds in delta, alpha, beta, and gamma bands and in the theta frequency band for the right seed (see also the supplementary materials for results on ROIs). Only in the case of theta and left seed occurs a negative shift as compared to the theoretical null distribution. The evaluation of the topography of *t*-value distributions indicated widespread effects, involving regions remote from the anode and cathode (Figure 5).

## 4. Discussion

In this study, we have combined noninvasive brain stimulation and high-resolution magnetoencephalography (MEG) and provided evidence that bilateral tDCS reshapes resting-state brain networks.

During the last 20 years, noninvasive brain stimulation techniques have been exploited for the investigation and treatment of a number of neurological disorders because of their ability of inducing LTP- and LTD-like plasticity phenomena.

Plasticity induction has been usually measured as changes of brain excitability [10, 38]. However, previous studies have demonstrated that both noninvasive brain stimulation and cortical plasticity are also associated with changes of brain rhythms and synchronization [39–41]. Conversely, robust and consistent evidence suggests that also neurological conditions translate into changes of brain activity and synchronization [41]. For instance, an impairment of cortical synchronization is often found at the first stages of neuropsychiatric conditions, up to the healthy subjects who own an increased risk [42–44]. More importantly, clinical recovery seems to be associated with changes of cortical rhythms and synchronization [45–47].

Our study was performed with a translational perspective and aimed at investigating the effects of bilateral tDCS on the healthy brain to better tailor treatment of neurological patients.

Both measures of brain activity and connectivity showed a significant tDCS-related modulation after real tDCS as compared to sham. Brain activity, as assessed by Power spectral density, is an indirect measure of cortical synchronization/desynchronization. Previous investigations of the tDCS effects on PSD have been largely performed with EEG and have provided conflicting results, in terms of the frequency bands and brain regions affected by the stimulation [48–55]. Our MEG study provides higher accuracy in the detection and modeling of cortical activity and supported a consistent and reduction of alpha, beta, and gamma power of the left frontal regions, ipsilateral to the anode and contralateral to the cathode. Changes in power can be related to an enhancement of the brain activation [56, 57] and are often found in neurological disorders, such as stroke [58] and epilepsy [41]. For these and other neurological disorders, tDCS application, rather being tailored on the base of the effect produced on brain excitability alone [8, 9], might benefit from taking into consideration the effects on brain activity and connectivity.

PSD results also confirm two additional relevant aspects in a translational perspective: (a) tDCS effects on cortical activity (PSD) depend upon the position of the anode and cathode [48] and (b) stronger effects are not necessarily confined under the region of stimulation but can involve remote regions. Our results on tDCS remote effects are in agreement with previous evidence from other approaches. Remote effects of brain stimulation have been indeed demonstrated for several measures of brain function, such as brain activity [15], cortical excitability [59, 60], hemodynamic activity and connectivity as measured by BOLD signal [61], and behavioral measures [62].

The connectivity analysis was performed on the entire cortex, taking into account the two seeds located in the sensorimotor regions, under the cathode and anode. For both seeds and for all the frequency bands under investigations, we found a significant increase of the synchronization of tDCS-related cortical activity. Such effect was widespread and particularly evident in delta, alpha, beta, and gamma bands. PLV results displayed in Figure 5 (and also the ROI-based analysis reported in the supplementary material) suggest a more widespread, homogeneous, short- and long-range increase of connectivity, especially when considering the seed under the anode (+, increasing cortical excitability), and support the idea that the effects of the stimulation depend upon the interaction with networks rather than brain areas [19].

Very recently, and in agreement with our results, other groups have reported a global increase of EEG synchronization after tDCS [63] and diffuse changes of connectivity in post-tDCS fMRI [64]. Vecchio and collaborators have suggested that tDCS effects on cortical coherence are polarity dependent, mainly involve alpha band, and are characterized by a connectivity increase [65]. Beyond the differences of experimental design, similar findings are supported by the investigation of Mancini and colleagues [66].

The findings of this study arise from a very specific setting, characterized by bilateral stimulation performed on healthy subjects. In a translational perspective, it will be necessary to investigate how tDCS-related activity and connectivity changes are influenced by several individual factors, such as genetic pattern [67, 68], gender [69], spontaneous fluctuations of cortical activity and excitability [70] and, especially in patients, the effect of brain lesions [11], cortical degeneration [71], and the influence of medications [72]. It will also be crucial to address the time dynamics of the effects on brain networks, in order to tune appropriately the duration, frequency, and dose of tDCS.

## 5. Conclusions

tDCS is a noninvasive brain stimulation approach which is becoming very popular and currently exploited to treat neurological disorders. We have demonstrated that bilateral tDCS (left anode and right cathode) reduces left alpha, beta, and gamma power and increases global connectivity in delta, alpha, beta, and gamma frequencies, in a diffuse fashion. We have also demonstrated that, beyond the well-known effects on brain excitability, tDCS reshapes resting-state brain networks. This information can be of help to understand the plasticity phenomena induced by noninvasive brain stimulation and can be exploited to tailor the therapeutic intervention in patients affected by neurological conditions.

## Supplementary Materials

Supplementary Materials The supplementary materials contain additional analyses conducted at ROI level, to test the regional effect of tDCS on PSD and on PLV measures. Eleven bilateral ROIs covering the whole cortex surface were selected from the automatic parcellation of Destrieux Atlas [1], as implemented in the Brainstorm Toolbox [2]. For each ROI a paired *t*-test was performed, comparing the effect of real tDCS with the effect of sham tDCS. The effect was calculated as percentage increase in the poststimulation as compared to the pre [(post − pre/pre) ∗ 100]. Importantly, the information provided in these tables is different from the information of the global analysis included in the manuscript. While the analysis in the manuscript tests whether there is a global effect of tDCS on the measures, these tables show if there are specific effects in the average value of the measure calculated for each specific ROI. Each table reports (1) the name of the ROI, the “L” or “R” at the end of the name which indicate if the ROI was in the left or right hemisphere; (2) the degrees of freedom of the test; (3) the *t*-value; (4) the effect size, calculated as Cohen's d; (5) the *p* value; (6) the significance of the *p* value with asterisks “∗” denoting values below 0.05; (7) the direction of the significant difference, with “>” indicating “Real > Sham” and “<” indicating “Real < Sham”; (8) the mean values of the effect in the real condition (SD enclosed in parenthesis); (9) the mean values for the effect in the sham condition (SD enclosed in parenthesis).
