# Supplementary material for: Bilateral Transcranial Direct Current Stimulation Reshapes Resting-State Brain Networks: A Magnetoencephalography Assessment
Source: Neural Plast. 2018 Jan 11;2018:2782804. doi: 10.1155/2018/2782804 (PMC5821992; doi:10.1155/2018/2782804)
Supplement: Supplementary Materials — The supplementary materials contain additional analyses conducted at ROI level, to test the regional effect of tDCS on PSD and on PLV measures. Eleven bilateral ROIs covering the whole cortex surface were selected from the automatic parcellation of Destrieux Atlas [1], as implemented in the Brainstorm Toolbox [2]. For each ROI a paired t-test was performed, comparing the effect of real tDCS with the effect of sham tDCS. The effect was calculated as percentage increase in the poststimulation as compared to the pre [(post − pre/pre) ∗ 100]. Importantly, the information provided in these tables is different from the information of the global analysis included in the manuscript. While the analysis in the manuscript tests whether there is a global effect of tDCS on the measures, these tables show if there are specific effects in the average value of the measure calculated for each specific ROI. Each table reports (1) the name of the ROI, the “L” or “R” at the end of the name which indicate if the ROI was in the left or right hemisphere; (2) the degrees of freedom of the test; (3) the t-value; (4) the effect size, calculated as Cohen's d; (5) the p value; (6) the significance of the p value with asterisks “∗” denoting values below 0.05; (7) the direction of the significant difference, with “>” indicating “Real > Sham” and “<” indicating “Real < Sham”; (8) the mean values of the effect in the real condition (SD enclosed in parenthesis); (9) the mean values for the effect in the sham condition (SD enclosed in parenthesis). [file 2782804.f1.docx]

**Supplementary Materials**

This supplementary material contains additional analyses conducted at ROI level, to test the regional effect of tDCS on PSD and on PLV measures. Eleven bilateral ROIs covering the whole cortex surface were selected from the automatic parcellation of Destrieux Atlas[1], as implemented in the Brainstorm Toolbox[2]. For each ROI a paired t-test was performed comparing the effect of Real tDCS with the effect of Sham tDCS. The effect was calculated as percentage increase in the post stimulation as compared to the pre [ (post – pre/ pre) * 100].

Importantly, the information provided in this tables is different from the information of the global analysis included in the manuscript. While the analysis in the manuscript tests whether there is a *global* effect of tDCS on the measures, these tables show if there is a specific effects in the *average* value of the measure calculated for each specific ROIs.

Each table reports: 1) the name of the ROI, the ‘L’ or ‘R’ at the end of the name indicate if the ROI was in the Left or Right hemisphere; 2) the degrees of freedom of the test; 3) the t-value; 4) the effect size, calculated as Cohen’s d; 5) the p-value; 6) the significance of the p-value with asterisks ‘*’ denoting values below 0.05; 7) the direction of the significant difference, with ‘>’ indicating ‘Real > Sham’, and ‘<’ indicating ‘Real < Sham’; 8) The mean values of the effect in the Real condition (SD enclosed in parenthesis); 9) The mean values for the effect the Sham condition (SD enclosed in parenthesis).

**PSD – delta**

| ROI | df | t | Cohen's d | p | sig | direction | Mean Real (SD) | Mean Sham (SD) |
| --- | --- | --- | --- | --- | --- | --- | --- | --- |
| caudalmiddlefrontal.L | 14 | -1,5 | -0,53 | 0,15 |  |  | -7.81 (44.43) | 30.62 (91.33) |
| caudalmiddlefrontal.R | 14 | -1,2 | -0,38 | 0,26 |  |  | -1.5 (59.69) | 28.5 (91.98) |
| inferiorparietal.L | 14 | 1,1 | 0,3 | 0,3 |  |  | 11.16 (37.99) | 1.5 (18.51) |
| inferiorparietal.R | 14 | 0,16 | 0,059 | 0,87 |  |  | 13.85 (32.61) | 11.72 (39.3) |
| lateraloccipital.L | 14 | 1,3 | 0,34 | 0,22 |  |  | 35.47 (73.9) | 13.59 (50.26) |
| lateraloccipital.R | 14 | 0,29 | 0,11 | 0,77 |  |  | 34.5 (72.19) | 25.13 (95.88) |
| middletemporal.L | 14 | -0,23 | -0,021 | 0,82 |  |  | 24.03 (81.62) | 25.79 (70.01) |
| middletemporal.R | 14 | -0,85 | -0,35 | 0,41 |  |  | 13.22 (35.06) | 101.6 (392.53) |
| postcentral.L | 14 | -1,2 | -0,34 | 0,24 |  |  | -6.26 (37) | 5.04 (27.19) |
| postcentral.R | 14 | -0,77 | -0,25 | 0,46 |  |  | 5.49 (54.4) | 20.27 (63.06) |
| precentral.L | 14 | -1,7 | -0,48 | 0,12 |  |  | -7.93 (38.81) | 11.7 (42.61) |
| precentral.R | 14 | -0,93 | -0,31 | 0,37 |  |  | 2.48 (54.28) | 23.61 (78.18) |
| rostralmiddlefrontal.L | 14 | -1,4 | -0,34 | 0,19 |  |  | 6.43 (36.48) | 51.51 (143.77) |
| rostralmiddlefrontal.R | 14 | -0,95 | -0,37 | 0,36 |  |  | 6.04 (47.47) | 41.16 (128.1) |
| superiorfrontal.L | 14 | -1,4 | -0,42 | 0,2 |  |  | -3.55 (43.78) | 18.26 (59.16) |
| superiorfrontal.R | 14 | -1,2 | -0,36 | 0,26 |  |  | -2.92 (50.4) | 18.16 (64.54) |
| superiorparietal.L | 14 | 0,39 | 0,13 | 0,7 |  |  | 8.08 (39.06) | 3.68 (24.97) |
| superiorparietal.R | 14 | 0,89 | 0,22 | 0,39 |  |  | 18.6 (57.84) | 7.57 (33.86) |
| superiortemporal.L | 14 | -1,3 | -0,13 | 0,22 |  |  | 9.78 (54.11) | 16.9 (48.95) |
| superiortemporal.R | 14 | -0,91 | -0,37 | 0,38 |  |  | 6.32 (28.4) | 72.47 (273.25) |
| supramarginal.L | 14 | -0,56 | -0,19 | 0,58 |  |  | -2.97 (26.44) | 1.57 (19.68) |
| supramarginal.R | 14 | -0,69 | -0,23 | 0,5 |  |  | 6.15 (46.03) | 16.78 (47) |

**PSD – theta**

| ROI | df | t | Cohen's d | p | sig | direction | Mean Real (SD) | Mean Sham (SD) |
| --- | --- | --- | --- | --- | --- | --- | --- | --- |
| caudalmiddlefrontal.L | 14 | -1,9 | -0,55 | 0,072 |  |  | -7.88 (45.28) | 15.75 (40.16) |
| caudalmiddlefrontal.R | 14 | -0,79 | -0,2 | 0,44 |  |  | 1.55 (64.36) | 13.35 (50.07) |
| inferiorparietal.L | 14 | 0,5 | 0,12 | 0,62 |  |  | 17.11 (56.74) | 11.13 (27.35) |
| inferiorparietal.R | 14 | 0,18 | 0,069 | 0,86 |  |  | 16.7 (38.58) | 14.04 (38.12) |
| lateraloccipital.L | 14 | 0,75 | 0,18 | 0,47 |  |  | 34.57 (99.78) | 18.7 (52.51) |
| lateraloccipital.R | 14 | 0,71 | 0,28 | 0,49 |  |  | 42.52 (104.19) | 19.17 (55.05) |
| middletemporal.L | 14 | -1,3 | -0,15 | 0,21 |  |  | 13.24 (39.89) | 25.87 (69.1) |
| middletemporal.R | 14 | -0,66 | -0,28 | 0,52 |  |  | 21.27 (51.57) | 58.55 (196.42) |
| postcentral.L | 14 | -1,4 | -0,4 | 0,17 |  |  | -6.3 (36.27) | 6.18 (23.12) |
| postcentral.R | 14 | -0,53 | -0,16 | 0,6 |  |  | 6.06 (57.92) | 14.17 (43.21) |
| precentral.L | 14 | -1,7 | -0,45 | 0,11 |  |  | -7.69 (41.44) | 9.13 (30.4) |
| precentral.R | 14 | -0,39 | -0,11 | 0,71 |  |  | 6.29 (61.28) | 12.27 (45.58) |
| rostralmiddlefrontal.L | 14 | -2,3 | -0,7 | 0,034 | * | < | -3.1 (30.77) | 24.46 (45.45) |
| rostralmiddlefrontal.R | 14 | -0,89 | -0,33 | 0,39 |  |  | 8.02 (46.29) | 25.53 (60.07) |
| superiorfrontal.L | 14 | -1,4 | -0,4 | 0,17 |  |  | -2.4 (44.64) | 14.37 (38.98) |
| superiorfrontal.R | 14 | -0,92 | -0,24 | 0,37 |  |  | 2.12 (55.58) | 14.27 (42.67) |
| superiorparietal.L | 14 | 0,15 | 0,056 | 0,88 |  |  | 10.45 (43.21) | 8.41 (28.22) |
| superiorparietal.R | 14 | 0,6 | 0,17 | 0,56 |  |  | 20.73 (63.58) | 11.62 (36.69) |
| superiortemporal.L | 14 | -1,9 | -0,39 | 0,082 |  |  | 1.5 (20.08) | 16.31 (40.39) |
| superiortemporal.R | 14 | -0,79 | -0,33 | 0,44 |  |  | 14.76 (40.72) | 47.78 (145.42) |
| supramarginal.L | 14 | -1,3 | -0,49 | 0,2 |  |  | -2.04 (19.48) | 7.29 (18.44) |
| supramarginal.R | 14 | -0,78 | -0,25 | 0,45 |  |  | 6.42 (51.55) | 18.49 (44.92) |

**PSD – alpha**

| ROI | df | t | Cohen's d | p | sig | direction | Mean Real (SD) | Mean Sham (SD) |
| --- | --- | --- | --- | --- | --- | --- | --- | --- |
| caudalmiddlefrontal.L | 14 | -0,64 | -0,21 | 0,54 |  |  | 10.34 (60.21) | 20.71 (36.02) |
| caudalmiddlefrontal.R | 14 | -0,22 | -0,058 | 0,83 |  |  | 23.82 (92.55) | 28.52 (60.62) |
| inferiorparietal.L | 14 | 0,7 | 0,27 | 0,5 |  |  | 44.69 (67.79) | 28.93 (49.22) |
| inferiorparietal.R | 14 | 1 | 0,24 | 0,31 |  |  | 65.19 (74.4) | 48.04 (65.47) |
| lateraloccipital.L | 14 | 1,5 | 0,36 | 0,17 |  |  | 79.96 (150.18) | 32.22 (65) |
| lateraloccipital.R | 14 | 1,7 | 0,48 | 0,12 |  |  | 113.06 (191.46) | 35.31 (39.33) |
| middletemporal.L | 14 | 0,099 | 0,022 | 0,92 |  |  | 37.86 (47.99) | 36.28 (78.48) |
| middletemporal.R | 14 | 1,3 | 0,33 | 0,21 |  |  | 54.5 (73.24) | 33.08 (44.14) |
| postcentral.L | 14 | -0,2 | -0,071 | 0,84 |  |  | 21.21 (79.82) | 25.82 (43.84) |
| postcentral.R | 14 | 0,54 | 0,13 | 0,6 |  |  | 61.41 (153.9) | 43.36 (79.29) |
| precentral.L | 14 | -0,27 | -0,09 | 0,79 |  |  | 17.9 (77.52) | 23.6 (41.79) |
| precentral.R | 14 | 0,26 | 0,066 | 0,8 |  |  | 43.41 (129.24) | 35.93 (69.97) |
| rostralmiddlefrontal.L | 14 | -0,6 | -0,16 | 0,56 |  |  | 19.58 (37.65) | 24.95 (26.86) |
| rostralmiddlefrontal.R | 14 | 0,39 | 0,12 | 0,7 |  |  | 31.28 (65.58) | 24.71 (32.8) |
| superiorfrontal.L | 14 | -0,12 | -0,039 | 0,91 |  |  | 15.86 (55.24) | 17.68 (33.49) |
| superiorfrontal.R | 14 | -0,054 | -0,016 | 0,96 |  |  | 21.26 (70.97) | 22.2 (42.23) |
| superiorparietal.L | 14 | 0,65 | 0,21 | 0,52 |  |  | 52.34 (108.8) | 33.63 (61.88) |
| superiorparietal.R | 14 | 1 | 0,21 | 0,32 |  |  | 87.98 (176.68) | 52.56 (93.88) |
| superiortemporal.L | 14 | 0,055 | 0,016 | 0,96 |  |  | 27.84 (37.83) | 27.14 (47.08) |
| superiortemporal.R | 14 | 0,83 | 0,27 | 0,42 |  |  | 43.65 (50.6) | 31.6 (38.05) |
| supramarginal.L | 14 | -0,13 | -0,055 | 0,9 |  |  | 18.95 (44.07) | 21.14 (35.6) |
| supramarginal.R | 14 | 0,53 | 0,16 | 0,61 |  |  | 51.71 (85.06) | 39.8 (61.13) |

**PSD – beta**

| ROI | df | t | Cohen's d | p | sig | direction | Mean Real (SD) | Mean Sham (SD) |
| --- | --- | --- | --- | --- | --- | --- | --- | --- |
| caudalmiddlefrontal.L | 14 | -1,3 | -0,29 | 0,21 |  |  | -6.79 (39.27) | 3.52 (27.35) |
| caudalmiddlefrontal.R | 14 | -0,3 | -0,065 | 0,77 |  |  | 1.96 (59.82) | 5.49 (40.57) |
| inferiorparietal.L | 14 | -0,0082 | -0,0017 | 0,99 |  |  | 15.08 (43.6) | 15.15 (34.72) |
| inferiorparietal.R | 14 | 0,48 | 0,18 | 0,64 |  |  | 18.06 (21.44) | 13.89 (25.32) |
| lateraloccipital.L | 14 | 1,5 | 0,28 | 0,17 |  |  | 41.98 (89.2) | 18.64 (57.71) |
| lateraloccipital.R | 14 | 1,6 | 0,45 | 0,14 |  |  | 50 (92.11) | 15.12 (23.74) |
| middletemporal.L | 14 | -0,64 | -0,069 | 0,53 |  |  | 13.73 (30.91) | 18.26 (52.58) |
| middletemporal.R | 14 | 1,2 | 0,44 | 0,26 |  |  | 18.66 (25.59) | 9.28 (16.54) |
| postcentral.L | 14 | -1,4 | -0,39 | 0,2 |  |  | -2.61 (32.9) | 8.66 (22.66) |
| postcentral.R | 14 | -0,13 | -0,034 | 0,9 |  |  | 6.32 (51.25) | 7.85 (35.43) |
| precentral.L | 14 | -1,3 | -0,31 | 0,22 |  |  | -4.99 (39.53) | 5.96 (25.36) |
| precentral.R | 14 | -0,033 | -0,0075 | 0,97 |  |  | 5.25 (60.74) | 5.66 (37.96) |
| rostralmiddlefrontal.L | 14 | -2,2 | -0,46 | 0,048 | * | < | -1.18 (23.17) | 8.55 (17.79) |
| rostralmiddlefrontal.R | 14 | -0,43 | -0,11 | 0,68 |  |  | 4.57 (35.74) | 8.27 (27.93) |
| superiorfrontal.L | 14 | -1,1 | -0,28 | 0,29 |  |  | -4.54 (34.16) | 4.21 (25.93) |
| superiorfrontal.R | 14 | -0,71 | -0,15 | 0,49 |  |  | -0.69 (43.92) | 5.27 (32.31) |
| superiorparietal.L | 14 | -0,2 | -0,077 | 0,85 |  |  | 9.82 (33.28) | 12.11 (25.66) |
| superiorparietal.R | 14 | 0,27 | 0,081 | 0,79 |  |  | 18.34 (45.66) | 15.12 (31.89) |
| superiortemporal.L | 14 | -1 | -0,17 | 0,31 |  |  | 7.52 (20.62) | 14.12 (37.85) |
| superiortemporal.R | 14 | 0,64 | 0,23 | 0,53 |  |  | 15.67 (24.07) | 10.11 (23.76) |
| supramarginal.L | 14 | -1,3 | -0,34 | 0,22 |  |  | 1.9 (19.9) | 10.77 (29.38) |
| supramarginal.R | 14 | 0,018 | 0,0057 | 0,99 |  |  | 11.98 (31.62) | 11.8 (33.84) |

**PSD – gamma**

| ROI | df | t | Cohen's d | p | sig | direction | Mean Real (SD) | Mean Sham (SD) |
| --- | --- | --- | --- | --- | --- | --- | --- | --- |
| caudalmiddlefrontal.L | 14 | -1,9 | -0,79 | 0,077 |  |  | -4.35 (11.05) | 3.21 (7.92) |
| caudalmiddlefrontal.R | 14 | 0,38 | 0,1 | 0,71 |  |  | 2.27 (27.89) | -0.39 (22.35) |
| inferiorparietal.L | 14 | -0,55 | -0,1 | 0,59 |  |  | 7.51 (27.78) | 10.22 (18.4) |
| inferiorparietal.R | 14 | 0,96 | 0,38 | 0,35 |  |  | 11.41 (22.6) | 4.9 (9.99) |
| lateraloccipital.L | 14 | 1 | 0,19 | 0,33 |  |  | 13.35 (26.23) | 8.64 (14.92) |
| lateraloccipital.R | 14 | 1,5 | 0,52 | 0,16 |  |  | 19.04 (30.74) | 6.83 (10.87) |
| middletemporal.L | 14 | -1,6 | -0,15 | 0,13 |  |  | 12.54 (32.64) | 18.26 (37.35) |
| middletemporal.R | 14 | 0,33 | 0,039 | 0,75 |  |  | 11.15 (25.62) | 10.18 (24.16) |
| postcentral.L | 14 | -1,8 | -0,5 | 0,087 |  |  | -1.04 (12) | 5.61 (14.14) |
| postcentral.R | 14 | 0,28 | 0,081 | 0,78 |  |  | 1.64 (18.64) | 0.06 (20.08) |
| precentral.L | 14 | -2,3 | -0,85 | 0,035 | * | < | -4.4 (10.23) | 4.58 (10.86) |
| precentral.R | 14 | 0,29 | 0,083 | 0,77 |  |  | 1.93 (28.55) | -0.17 (20.84) |
| rostralmiddlefrontal.L | 14 | -0,78 | -0,28 | 0,45 |  |  | 0.49 (13.39) | 3.83 (10.09) |
| rostralmiddlefrontal.R | 14 | -0,55 | -0,2 | 0,59 |  |  | 2.44 (16.42) | 5.43 (13.66) |
| superiorfrontal.L | 14 | -1,3 | -0,43 | 0,23 |  |  | -2.41 (14.35) | 2.96 (9.89) |
| superiorfrontal.R | 14 | -0,61 | -0,18 | 0,55 |  |  | 0.09 (17.79) | 2.85 (12.57) |
| superiorparietal.L | 14 | -0,12 | -0,041 | 0,91 |  |  | 4.47 (12.74) | 4.91 (7.64) |
| superiorparietal.R | 14 | 0,91 | 0,34 | 0,38 |  |  | 7.56 (15.59) | 2.92 (11.06) |
| superiortemporal.L | 14 | -2,1 | -0,21 | 0,059 |  |  | 9.23 (29.3) | 18.94 (41.64) |
| superiortemporal.R | 14 | 0,11 | 0,025 | 0,92 |  |  | 8.64 (14.94) | 8.25 (15.13) |
| supramarginal.L | 14 | -1,5 | -0,27 | 0,15 |  |  | 2.85 (23.17) | 8.99 (22.38) |
| supramarginal.R | 14 | 0,82 | 0,28 | 0,43 |  |  | 8.63 (24.72) | 2.39 (18.78) |

**PLV – Left Seed – Delta**

| ROI | df | t | Cohen's d | p | sig | direction | Mean Real (SD) | Mean Sham (SD) |
| --- | --- | --- | --- | --- | --- | --- | --- | --- |
| caudalmiddlefrontal.L | 14 | -0,3 | -0,13 | 0,77 |  |  | 29.08 (40.08) | 33.93 (36.44) |
| caudalmiddlefrontal.R | 14 | 1,4 | 0,56 | 0,2 |  |  | 56.58 (64.42) | 30 (26.24) |
| inferiorparietal.L | 14 | 0,53 | 0,24 | 0,6 |  |  | 42.15 (27.73) | 33.42 (43.4) |
| inferiorparietal.R | 14 | 1,8 | 0,57 | 0,087 |  |  | 90.02 (83.12) | 47.97 (60.35) |
| lateraloccipital.L | 14 | 0,63 | 0,24 | 0,54 |  |  | 70.65 (40.51) | 60.21 (47.31) |
| lateraloccipital.R | 14 | 0,57 | 0,2 | 0,58 |  |  | 76.75 (53.74) | 66.92 (46.35) |
| middletemporal.L | 14 | 1,2 | 0,52 | 0,24 |  |  | 52.42 (40.7) | 31.36 (40.56) |
| middletemporal.R | 14 | 0,065 | 0,025 | 0,95 |  |  | 57.41 (47.3) | 56.19 (51.33) |
| postcentral.L | 14 | 0,037 | 0,012 | 0,97 |  |  | 28.12 (26.25) | 27.78 (31.66) |
| postcentral.R | 14 | 1,9 | 0,78 | 0,08 |  |  | 83.06 (77.71) | 31.84 (51.98) |
| precentral.L | 14 | -0,78 | -0,26 | 0,45 |  |  | 22 (13.39) | 31.17 (45.72) |
| precentral.R | 14 | 2,1 | 0,82 | 0,057 |  |  | 66.39 (55.41) | 27.2 (39.69) |
| rostralmiddlefrontal.L | 14 | 1,7 | 0,49 | 0,11 |  |  | 71.45 (57.22) | 47.38 (34.48) |
| rostralmiddlefrontal.R | 14 | 0,12 | 0,047 | 0,9 |  |  | 58.73 (45.76) | 56.41 (53.45) |
| superiorfrontal.L | 14 | 1,9 | 0,8 | 0,076 |  |  | 51.26 (35.63) | 24.2 (31.65) |
| superiorfrontal.R | 14 | 2,4 | 0,86 | 0,03 | * | > | 66.46 (38.08) | 37.8 (27.3) |
| superiorparietal.L | 14 | 1,3 | 0,46 | 0,21 |  |  | 55.47 (40.63) | 38.92 (30.58) |
| superiorparietal.R | 14 | 1,7 | 0,55 | 0,11 |  |  | 128.37 (153.82) | 59.84 (74.12) |
| superiortemporal.L | 14 | 0,73 | 0,19 | 0,48 |  |  | 49.73 (42.57) | 41.1 (45.97) |
| superiortemporal.R | 14 | 1,9 | 0,69 | 0,076 |  |  | 80.37 (74.25) | 37.44 (46.47) |
| supramarginal.L | 14 | 1,2 | 0,37 | 0,24 |  |  | 27.17 (22.88) | 17.21 (30.6) |
| supramarginal.R | 14 | 0,27 | 0,099 | 0,79 |  |  | 75.09 (81.21) | 64.69 (123.72) |

**PLV – Left Seed – Theta**

| ROI | df | t | Cohen's d | p | sig | direction | Mean Real (SD) | Mean Sham (SD) |
| --- | --- | --- | --- | --- | --- | --- | --- | --- |
| caudalmiddlefrontal.L | 14 | -0,57 | -0,17 | 0,58 |  |  | 14.77 (44.35) | 21.24 (26.1) |
| caudalmiddlefrontal.R | 14 | -0,92 | -0,27 | 0,37 |  |  | 53.03 (101.38) | 77.82 (82.31) |
| inferiorparietal.L | 14 | 0,46 | 0,13 | 0,66 |  |  | 51.23 (63.05) | 43.26 (62.35) |
| inferiorparietal.R | 14 | -0,9 | -0,28 | 0,38 |  |  | 45.43 (63.3) | 62.91 (61.86) |
| lateraloccipital.L | 14 | -0,74 | -0,26 | 0,47 |  |  | 53.02 (54.13) | 67.89 (60.04) |
| lateraloccipital.R | 14 | -1 | -0,39 | 0,33 |  |  | 44.51 (61.76) | 67.75 (56.96) |
| middletemporal.L | 14 | -0,65 | -0,21 | 0,52 |  |  | 40 (65.95) | 52.38 (48.56) |
| middletemporal.R | 14 | -0,14 | -0,043 | 0,89 |  |  | 69.37 (89.81) | 72.71 (59.34) |
| postcentral.L | 14 | -1,5 | -0,49 | 0,15 |  |  | 10.59 (29.53) | 23.06 (19.74) |
| postcentral.R | 14 | -1,5 | -0,5 | 0,16 |  |  | 33.6 (59.47) | 160.57 (337.73) |
| precentral.L | 14 | -1,2 | -0,45 | 0,24 |  |  | 13.28 (24.76) | 23.38 (20.21) |
| precentral.R | 14 | -0,65 | -0,21 | 0,53 |  |  | 42.45 (53.42) | 52.35 (36.82) |
| rostralmiddlefrontal.L | 14 | -1 | -0,25 | 0,34 |  |  | 40.81 (58.98) | 54.08 (45.56) |
| rostralmiddlefrontal.R | 14 | -1,2 | -0,42 | 0,25 |  |  | 47.98 (67.87) | 77.55 (74.33) |
| superiorfrontal.L | 14 | -0,38 | -0,099 | 0,71 |  |  | 33.71 (49.45) | 39.1 (58.53) |
| superiorfrontal.R | 14 | -0,52 | -0,18 | 0,61 |  |  | 43.16 (52.62) | 53.43 (61.57) |
| superiorparietal.L | 14 | 0,71 | 0,22 | 0,49 |  |  | 42.27 (55.24) | 32.15 (32.94) |
| superiorparietal.R | 14 | -0,057 | -0,015 | 0,96 |  |  | 54.18 (73.22) | 55.13 (50.73) |
| superiortemporal.L | 14 | -0,012 | -0,0038 | 0,99 |  |  | 35.17 (55.98) | 35.34 (26.31) |
| superiortemporal.R | 14 | 0,12 | 0,038 | 0,91 |  |  | 73.08 (84.13) | 70.05 (75.26) |
| supramarginal.L | 14 | -0,8 | -0,16 | 0,44 |  |  | 20.66 (27.58) | 25.03 (27.56) |
| supramarginal.R | 14 | -1,8 | -0,61 | 0,09 |  |  | 39.22 (76.6) | 85.24 (73.63) |

**PLV – Left Seed – Alpha**

| ROI | df | t | Cohen's d | p | sig | direction | Mean Real (SD) | Mean Sham (SD) |
| --- | --- | --- | --- | --- | --- | --- | --- | --- |
| caudalmiddlefrontal.L | 14 | -0,22 | -0,085 | 0,83 |  |  | 31.55 (49.25) | 35.36 (39.87) |
| caudalmiddlefrontal.R | 14 | -0,78 | -0,16 | 0,45 |  |  | 86.36 (60.85) | 112.9 (166.59) |
| inferiorparietal.L | 14 | 2,8 | 1 | 0,014 | * | > | 89.36 (68.89) | 34.05 (36.37) |
| inferiorparietal.R | 14 | 0,51 | 0,18 | 0,62 |  |  | 140.87 (125.75) | 116.46 (146.79) |
| lateraloccipital.L | 14 | 1,5 | 0,5 | 0,16 |  |  | 90.48 (78.61) | 52.67 (71.81) |
| lateraloccipital.R | 14 | 0,098 | 0,034 | 0,92 |  |  | 114.43 (90.26) | 110.74 (124.87) |
| middletemporal.L | 14 | 1,6 | 0,5 | 0,14 |  |  | 91.57 (77.9) | 56.52 (59.78) |
| middletemporal.R | 14 | -0,14 | -0,046 | 0,89 |  |  | 130.18 (124.22) | 138.07 (206.43) |
| postcentral.L | 14 | -0,28 | -0,1 | 0,78 |  |  | 34.21 (44.29) | 37.68 (19.4) |
| postcentral.R | 14 | 0,53 | 0,2 | 0,6 |  |  | 134.44 (126.97) | 108.54 (135.47) |
| precentral.L | 14 | -1,3 | -0,37 | 0,22 |  |  | 24.61 (21.53) | 33.28 (24.8) |
| precentral.R | 14 | 0,3 | 0,1 | 0,77 |  |  | 98.88 (58.63) | 89.54 (113.5) |
| rostralmiddlefrontal.L | 14 | 1,6 | 0,38 | 0,14 |  |  | 110.51 (110.49) | 73.69 (69.25) |
| rostralmiddlefrontal.R | 14 | 0,2 | 0,053 | 0,84 |  |  | 136.24 (115.85) | 129.09 (149.61) |
| superiorfrontal.L | 14 | 0,23 | 0,082 | 0,82 |  |  | 57.41 (59.19) | 52.99 (47.86) |
| superiorfrontal.R | 14 | 0,86 | 0,29 | 0,4 |  |  | 94.76 (100.28) | 69.94 (64.93) |
| superiorparietal.L | 14 | 1,7 | 0,59 | 0,11 |  |  | 70.64 (81.99) | 33.62 (31.32) |
| superiorparietal.R | 14 | 0,79 | 0,3 | 0,44 |  |  | 109.88 (86.64) | 83.08 (91.3) |
| superiortemporal.L | 14 | 1,9 | 0,55 | 0,079 |  |  | 88.79 (96.2) | 44.95 (29.99) |
| superiortemporal.R | 14 | 0,24 | 0,088 | 0,81 |  |  | 153.6 (147.1) | 138.16 (200.44) |
| supramarginal.L | 14 | 1,4 | 0,3 | 0,18 |  |  | 47 (41.52) | 35.6 (25.19) |
| supramarginal.R | 14 | 1,1 | 0,38 | 0,31 |  |  | 153.21 (127.57) | 104.42 (129.28) |

**PLV – Left Seed – Beta**

| ROI | df | t | Cohen's d | p | sig | direction | Mean Real (SD) | Mean Sham (SD) |
| --- | --- | --- | --- | --- | --- | --- | --- | --- |
| caudalmiddlefrontal.L | 14 | 0,89 | 0,32 | 0,39 |  |  | 48.8 (87.69) | 27.42 (35.99) |
| caudalmiddlefrontal.R | 14 | 1,3 | 0,41 | 0,21 |  |  | 77.06 (85.44) | 47.42 (54.16) |
| inferiorparietal.L | 14 | 1,8 | 0,45 | 0,089 |  |  | 54.7 (48.94) | 35.37 (25.9) |
| inferiorparietal.R | 14 | 0,93 | 0,34 | 0,37 |  |  | 196.59 (315.15) | 117.57 (98.38) |
| lateraloccipital.L | 14 | 0,16 | 0,055 | 0,87 |  |  | 57.32 (52.76) | 54.7 (41.43) |
| lateraloccipital.R | 14 | 1,3 | 0,48 | 0,22 |  |  | 112.89 (124.4) | 68.61 (42.89) |
| middletemporal.L | 14 | -0,32 | -0,13 | 0,76 |  |  | 40.39 (31.44) | 44.55 (32.07) |
| middletemporal.R | 14 | 1,3 | 0,52 | 0,23 |  |  | 148.19 (162.73) | 83.11 (77.61) |
| postcentral.L | 14 | 0,42 | 0,11 | 0,68 |  |  | 20.74 (20.23) | 18.19 (26.27) |
| postcentral.R | 14 | 0,94 | 0,33 | 0,37 |  |  | 98.78 (117.44) | 69.02 (49.37) |
| precentral.L | 14 | 0,09 | 0,022 | 0,93 |  |  | 17.28 (18.34) | 16.64 (32.24) |
| precentral.R | 14 | 0,94 | 0,25 | 0,36 |  |  | 89.4 (101.98) | 67.28 (53.81) |
| rostralmiddlefrontal.L | 14 | 0,72 | 0,19 | 0,48 |  |  | 60.63 (46.71) | 51.32 (50.59) |
| rostralmiddlefrontal.R | 14 | 0,69 | 0,23 | 0,5 |  |  | 86.69 (67.34) | 72.25 (57.32) |
| superiorfrontal.L | 14 | 1,1 | 0,43 | 0,27 |  |  | 36.24 (47.08) | 20.3 (22.98) |
| superiorfrontal.R | 14 | 0,8 | 0,22 | 0,44 |  |  | 60.17 (50.54) | 50.12 (37.6) |
| superiorparietal.L | 14 | 0,067 | 0,021 | 0,95 |  |  | 36.89 (34.94) | 36.22 (28.04) |
| superiorparietal.R | 14 | 1,1 | 0,42 | 0,28 |  |  | 131.81 (166.87) | 76.51 (84.96) |
| superiortemporal.L | 14 | 0,3 | 0,12 | 0,76 |  |  | 51.8 (58.87) | 45.76 (41.7) |
| superiortemporal.R | 14 | 0,35 | 0,14 | 0,73 |  |  | 131.87 (142.84) | 113.68 (107.36) |
| supramarginal.L | 14 | -0,59 | -0,18 | 0,56 |  |  | 25.51 (27.57) | 32.37 (44.62) |
| supramarginal.R | 14 | 0,57 | 0,23 | 0,58 |  |  | 130.03 (146.14) | 101.99 (95.42) |

**PLV – Left Seed – Gamma**

| ROI | df | t | Cohen's d | p | sig | direction | Mean Real (SD) | Mean Sham (SD) |
| --- | --- | --- | --- | --- | --- | --- | --- | --- |
| caudalmiddlefrontal.L | 14 | 1,2 | 0,54 | 0,26 |  |  | 69.53 (137.94) | 23.01 (23.92) |
| caudalmiddlefrontal.R | 14 | 0,14 | 0,055 | 0,89 |  |  | 67.28 (123.06) | 61.42 (88.97) |
| inferiorparietal.L | 14 | 0,33 | 0,13 | 0,75 |  |  | 38.88 (75.68) | 32 (15.55) |
| inferiorparietal.R | 14 | 0,71 | 0,31 | 0,49 |  |  | 112.14 (280.64) | 56.15 (50.86) |
| lateraloccipital.L | 14 | 0,35 | 0,15 | 0,73 |  |  | 53.81 (87.54) | 43.77 (42.89) |
| lateraloccipital.R | 14 | 0,87 | 0,32 | 0,4 |  |  | 139.04 (332.05) | 63.62 (54.88) |
| middletemporal.L | 14 | 0,79 | 0,37 | 0,44 |  |  | 76.64 (151.77) | 41.02 (32.53) |
| middletemporal.R | 14 | 0,14 | 0,059 | 0,89 |  |  | 52.9 (151.22) | 46.61 (46.41) |
| postcentral.L | 14 | 1,6 | 0,7 | 0,12 |  |  | 33.37 (59.02) | 6.08 (12.13) |
| postcentral.R | 14 | 1,1 | 0,45 | 0,31 |  |  | 80.17 (125.22) | 42.19 (30.07) |
| precentral.L | 14 | 1,4 | 0,55 | 0,18 |  |  | 38.67 (56.84) | 16.77 (13.27) |
| precentral.R | 14 | 0,71 | 0,27 | 0,49 |  |  | 61.52 (131.63) | 36.62 (23.55) |
| rostralmiddlefrontal.L | 14 | 1,1 | 0,48 | 0,3 |  |  | 130.54 (323.59) | 36.29 (33.7) |
| rostralmiddlefrontal.R | 14 | 0,66 | 0,28 | 0,52 |  |  | 62.41 (125.04) | 38.21 (34.24) |
| superiorfrontal.L | 14 | 0,99 | 0,45 | 0,34 |  |  | 70.28 (149.12) | 28.63 (22.69) |
| superiorfrontal.R | 14 | 0,99 | 0,45 | 0,34 |  |  | 57.76 (92.59) | 29.9 (26.43) |
| superiorparietal.L | 14 | 0,64 | 0,29 | 0,53 |  |  | 37.97 (76.68) | 23.64 (15.85) |
| superiorparietal.R | 14 | 1,1 | 0,46 | 0,29 |  |  | 113.96 (266.01) | 35.11 (26.81) |
| superiortemporal.L | 14 | 0,83 | 0,37 | 0,42 |  |  | 68.28 (152.11) | 32.34 (28.19) |
| superiortemporal.R | 14 | 0,16 | 0,069 | 0,88 |  |  | 57.92 (130.31) | 51.41 (52.38) |
| supramarginal.L | 14 | 1,4 | 0,51 | 0,18 |  |  | 26.93 (38.18) | 10.9 (22.9) |
| supramarginal.R | 14 | 0,71 | 0,31 | 0,49 |  |  | 107.45 (235.98) | 60.19 (47.31) |

**PLV – Right Seed – Delta**

| ROI | df | t | Cohen's d | p | sig | direction | Mean Real (SD) | Mean Sham (SD) |
| --- | --- | --- | --- | --- | --- | --- | --- | --- |
| caudalmiddlefrontal.L | 14 | 1,1 | 0,35 | 0,29 |  |  | 79.55 (58.04) | 56.92 (68.95) |
| caudalmiddlefrontal.R | 14 | 2,6 | 0,8 | 0,022 | * | > | 66.42 (79.88) | 15.04 (15.43) |
| inferiorparietal.L | 14 | 2,6 | 1,1 | 0,02 | * | > | 124.36 (69.24) | 56.7 (57.44) |
| inferiorparietal.R | 14 | 0,63 | 0,2 | 0,54 |  |  | 63.01 (64.29) | 51.5 (50.41) |
| lateraloccipital.L | 14 | 3,6 | 1,3 | 0,0027 | * | > | 113.65 (58.31) | 50.87 (33.36) |
| lateraloccipital.R | 14 | 2,2 | 0,97 | 0,042 | * | > | 145.59 (146.13) | 46.75 (48.33) |
| middletemporal.L | 14 | 2,1 | 0,87 | 0,056 |  |  | 105.68 (88.69) | 47.37 (40.98) |
| middletemporal.R | 14 | 1,7 | 0,5 | 0,11 |  |  | 77.37 (87.32) | 38.43 (63.82) |
| postcentral.L | 14 | 4,1 | 1,4 | 0,001 | * | > | 90.89 (43.06) | 41.11 (23.68) |
| postcentral.R | 14 | 3,4 | 1,1 | 0,0046 | * | > | 54.77 (42.12) | 18.68 (14.32) |
| precentral.L | 14 | 4 | 1,4 | 0,0014 | * | > | 94.38 (48.42) | 37.45 (34.17) |
| precentral.R | 14 | 3 | 0,67 | 0,01 | * | > | 41.61 (28.19) | 24.52 (18.73) |
| rostralmiddlefrontal.L | 14 | 2,1 | 0,72 | 0,059 |  |  | 115.1 (104.73) | 56.22 (45.66) |
| rostralmiddlefrontal.R | 14 | 3,4 | 1,2 | 0,004 | * | > | 107.7 (83.54) | 31.81 (28.33) |
| superiorfrontal.L | 14 | 2,7 | 0,88 | 0,018 | * | > | 91.3 (81.46) | 34.17 (36.23) |
| superiorfrontal.R | 14 | 3,3 | 1,1 | 0,0058 | * | > | 86.8 (72.14) | 23.27 (33.12) |
| superiorparietal.L | 14 | 3,6 | 1,3 | 0,0027 | * | > | 140.99 (93.97) | 43.94 (43.66) |
| superiorparietal.R | 14 | 2,4 | 0,93 | 0,03 | * | > | 84.49 (75.68) | 30.97 (33.17) |
| superiortemporal.L | 14 | 1,6 | 0,55 | 0,14 |  |  | 77.83 (59.97) | 49.24 (42.08) |
| superiortemporal.R | 14 | 1,6 | 0,6 | 0,14 |  |  | 114.35 (193.62) | 34.12 (29.1) |
| supramarginal.L | 14 | 1,8 | 0,77 | 0,091 |  |  | 89.4 (67.71) | 48.24 (37.54) |
| supramarginal.R | 14 | 1,5 | 0,55 | 0,16 |  |  | 88.07 (156.87) | 26.49 (34.15) |

**PLV – Right Seed – Theta**

| ROI | df | t | Cohen's d | p | sig | direction | Mean Real (SD) | Mean Sham (SD) |
| --- | --- | --- | --- | --- | --- | --- | --- | --- |
| caudalmiddlefrontal.L | 14 | 0,91 | 0,34 | 0,38 |  |  | 67.39 (106.22) | 39.95 (46.52) |
| caudalmiddlefrontal.R | 14 | 0,55 | 0,17 | 0,59 |  |  | 30.56 (48.21) | 22.96 (38.36) |
| inferiorparietal.L | 14 | 1,3 | 0,41 | 0,22 |  |  | 83.52 (79.21) | 56.43 (45.31) |
| inferiorparietal.R | 14 | 0,71 | 0,23 | 0,49 |  |  | 58.72 (98.84) | 40.21 (46.27) |
| lateraloccipital.L | 14 | 0,28 | 0,11 | 0,78 |  |  | 68.37 (72.02) | 61.91 (45.76) |
| lateraloccipital.R | 14 | 0,41 | 0,14 | 0,69 |  |  | 82.71 (115.28) | 69.25 (73.44) |
| middletemporal.L | 14 | 1,2 | 0,37 | 0,23 |  |  | 99.1 (92.93) | 70.98 (39.41) |
| middletemporal.R | 14 | 0,43 | 0,17 | 0,68 |  |  | 51.5 (74.65) | 41.14 (46.97) |
| postcentral.L | 14 | 0,85 | 0,33 | 0,41 |  |  | 70.03 (72.69) | 52.7 (21.83) |
| postcentral.R | 14 | -0,15 | -0,05 | 0,88 |  |  | 32.1 (36.53) | 33.73 (28.02) |
| precentral.L | 14 | -0,052 | -0,02 | 0,96 |  |  | 64.99 (66.7) | 66.26 (60.87) |
| precentral.R | 14 | -0,06 | -0,021 | 0,95 |  |  | 29.52 (34.41) | 30.21 (32.33) |
| rostralmiddlefrontal.L | 14 | 1,1 | 0,4 | 0,28 |  |  | 100.77 (132.56) | 60.92 (47.74) |
| rostralmiddlefrontal.R | 14 | 1,1 | 0,35 | 0,3 |  |  | 61.09 (67.91) | 40.97 (41.32) |
| superiorfrontal.L | 14 | 0,63 | 0,18 | 0,54 |  |  | 70.82 (74.01) | 59.45 (41.86) |
| superiorfrontal.R | 14 | 0,98 | 0,26 | 0,35 |  |  | 53.07 (55.76) | 40.62 (34.28) |
| superiorparietal.L | 14 | 0,99 | 0,36 | 0,34 |  |  | 107.67 (113.4) | 74.24 (65.04) |
| superiorparietal.R | 14 | 0,58 | 0,19 | 0,57 |  |  | 62.93 (98.34) | 48.36 (35.11) |
| superiortemporal.L | 14 | 1,1 | 0,39 | 0,31 |  |  | 94.43 (88.28) | 68.5 (34.87) |
| superiortemporal.R | 14 | 1 | 0,34 | 0,31 |  |  | 64.96 (97.44) | 37.58 (51.67) |
| supramarginal.L | 14 | 0,85 | 0,3 | 0,41 |  |  | 78.87 (96.98) | 54.54 (58.02) |
| supramarginal.R | 14 | 0,95 | 0,31 | 0,36 |  |  | 44.01 (80.36) | 24.19 (34.77) |

**PLV – Right Seed – alpha**

| ROI | df | t | Cohen's d | p | sig | direction | Mean Real (SD) | Mean Sham (SD) |
| --- | --- | --- | --- | --- | --- | --- | --- | --- |
| caudalmiddlefrontal.L | 14 | 2,2 | 0,69 | 0,049 | * | > | 229.32 (242.7) | 92.62 (124.5) |
| caudalmiddlefrontal.R | 14 | 2,1 | 0,79 | 0,053 |  |  | 108.97 (136.72) | 30.97 (35.79) |
| inferiorparietal.L | 14 | 2,8 | 1,1 | 0,013 | * | > | 191.92 (135.7) | 73.96 (75.6) |
| inferiorparietal.R | 14 | 1,9 | 0,63 | 0,072 |  |  | 157.79 (186.44) | 64.89 (69.98) |
| lateraloccipital.L | 14 | 2,2 | 0,88 | 0,041 | * | > | 199.35 (174.9) | 71.76 (109.24) |
| lateraloccipital.R | 14 | 1,9 | 0,73 | 0,078 |  |  | 191.2 (235.35) | 66.97 (71.82) |
| middletemporal.L | 14 | 2,2 | 0,69 | 0,041 | * | > | 205.86 (222.56) | 81.3 (84.66) |
| middletemporal.R | 14 | 1,7 | 0,61 | 0,12 |  |  | 162.87 (184.77) | 70.72 (105.56) |
| postcentral.L | 14 | 2,9 | 0,91 | 0,012 | * | > | 202.43 (190.18) | 64.15 (73.88) |
| postcentral.R | 14 | 1,6 | 0,52 | 0,14 |  |  | 71.04 (80.15) | 38.68 (28.75) |
| precentral.L | 14 | 3,3 | 0,94 | 0,0054 | * | > | 191.47 (159.6) | 66.71 (68.23) |
| precentral.R | 14 | 1,5 | 0,55 | 0,14 |  |  | 67.55 (103.01) | 25.55 (25.3) |
| rostralmiddlefrontal.L | 14 | 2,3 | 0,63 | 0,034 | * | > | 281.97 (303.22) | 118.12 (106.87) |
| rostralmiddlefrontal.R | 14 | 1,1 | 0,4 | 0,27 |  |  | 197.05 (224.24) | 120.08 (148.55) |
| superiorfrontal.L | 14 | 2,5 | 0,71 | 0,023 | * | > | 192.35 (192.91) | 76.82 (66.08) |
| superiorfrontal.R | 14 | 1,9 | 0,54 | 0,073 |  |  | 132.4 (134.95) | 71.17 (68.14) |
| superiorparietal.L | 14 | 2 | 0,77 | 0,064 |  |  | 194.51 (204.21) | 75.21 (88.8) |
| superiorparietal.R | 14 | 1,5 | 0,51 | 0,15 |  |  | 118.96 (177.67) | 49.21 (54.38) |
| superiortemporal.L | 14 | 1,5 | 0,52 | 0,16 |  |  | 235.07 (269.09) | 121.35 (149.22) |
| superiortemporal.R | 14 | 1,8 | 0,7 | 0,098 |  |  | 135.05 (180.98) | 46.37 (45.26) |
| supramarginal.L | 14 | 1,9 | 0,74 | 0,082 |  |  | 224.29 (253.85) | 85.03 (96.95) |
| supramarginal.R | 14 | 1,4 | 0,44 | 0,17 |  |  | 99.16 (103.71) | 62.15 (47.89) |

**PLV – Right Seed – beta**

| ROI | df | t | Cohen's d | p | sig | direction | Mean Real (SD) | Mean Sham (SD) |
| --- | --- | --- | --- | --- | --- | --- | --- | --- |
| caudalmiddlefrontal.L | 14 | 1,3 | 0,41 | 0,23 |  |  | 104.91 (134.46) | 61.01 (57.38) |
| caudalmiddlefrontal.R | 14 | 1 | 0,33 | 0,33 |  |  | 41.78 (53.1) | 25.88 (41.64) |
| inferiorparietal.L | 14 | 1,9 | 0,41 | 0,083 |  |  | 217.46 (348.43) | 64.54 (52.67) |
| inferiorparietal.R | 14 | 1,2 | 0,42 | 0,26 |  |  | 86.63 (93.59) | 56.26 (41.27) |
| lateraloccipital.L | 14 | 1,7 | 0,66 | 0,11 |  |  | 192.78 (208.53) | 90.48 (78.71) |
| lateraloccipital.R | 14 | 1,6 | 0,54 | 0,13 |  |  | 150.55 (204.12) | 64.98 (64.92) |
| middletemporal.L | 14 | 0,87 | 0,32 | 0,4 |  |  | 135.97 (138.31) | 98.33 (92.35) |
| middletemporal.R | 14 | 2 | 0,65 | 0,071 |  |  | 96.77 (99.31) | 44.27 (50.76) |
| postcentral.L | 14 | 1,8 | 0,55 | 0,098 |  |  | 113.93 (115.27) | 62.12 (52.67) |
| postcentral.R | 14 | 2 | 0,58 | 0,066 |  |  | 36.04 (41.81) | 15.92 (11.3) |
| precentral.L | 14 | 1,6 | 0,48 | 0,13 |  |  | 103.84 (109.09) | 61.04 (48.92) |
| precentral.R | 14 | 2,1 | 0,47 | 0,059 |  |  | 28.63 (34.13) | 13.9 (14.78) |
| rostralmiddlefrontal.L | 14 | 1,6 | 0,52 | 0,13 |  |  | 136.98 (160.36) | 71.58 (46.26) |
| rostralmiddlefrontal.R | 14 | 2,1 | 0,61 | 0,056 |  |  | 79.89 (69.59) | 43.02 (47.98) |
| superiorfrontal.L | 14 | 2,2 | 0,46 | 0,042 | * | > | 98.64 (122.35) | 38.54 (30.62) |
| superiorfrontal.R | 14 | 1,8 | 0,49 | 0,094 |  |  | 70.81 (90.17) | 33.15 (43.73) |
| superiorparietal.L | 14 | 1,2 | 0,4 | 0,23 |  |  | 124.84 (114.14) | 80.2 (106.35) |
| superiorparietal.R | 14 | 1,8 | 0,39 | 0,091 |  |  | 68.01 (81.31) | 37.61 (33.8) |
| superiortemporal.L | 14 | 0,64 | 0,23 | 0,53 |  |  | 118.49 (141.3) | 93.11 (70.69) |
| superiortemporal.R | 14 | 1,3 | 0,48 | 0,21 |  |  | 94.15 (124.06) | 48.46 (52.68) |
| supramarginal.L | 14 | 1,5 | 0,49 | 0,16 |  |  | 127.94 (141.03) | 72.66 (67.67) |
| supramarginal.R | 14 | 1,3 | 0,44 | 0,21 |  |  | 56.63 (66.73) | 32.69 (33.97) |

**PLV – Right Seed – gamma**

| ROI | df | t | Cohen's d | p | sig | direction | Mean Real (SD) | Mean Sham (SD) |
| --- | --- | --- | --- | --- | --- | --- | --- | --- |
| caudalmiddlefrontal.L | 14 | 2 | 0,59 | 0,07 |  |  | 103.49 (111.93) | 48.81 (57.36) |
| caudalmiddlefrontal.R | 14 | 1,4 | 0,58 | 0,17 |  |  | 77.84 (166.59) | 14.18 (18.66) |
| inferiorparietal.L | 14 | 1,1 | 0,39 | 0,28 |  |  | 81.45 (104.65) | 48.93 (49.45) |
| inferiorparietal.R | 14 | 1,5 | 0,6 | 0,16 |  |  | 81.35 (95.86) | 36.11 (51.51) |
| lateraloccipital.L | 14 | 1,1 | 0,38 | 0,3 |  |  | 88.03 (89.77) | 56.98 (72.75) |
| lateraloccipital.R | 14 | 1,4 | 0,49 | 0,18 |  |  | 117.83 (208.29) | 41.05 (58.46) |
| middletemporal.L | 14 | 0,78 | 0,28 | 0,45 |  |  | 96.58 (122.92) | 66.14 (92.21) |
| middletemporal.R | 14 | 1,1 | 0,42 | 0,27 |  |  | 77.29 (132.46) | 35.91 (42.66) |
| postcentral.L | 14 | 0,77 | 0,3 | 0,45 |  |  | 89.91 (113.76) | 62.58 (59.89) |
| postcentral.R | 14 | 1,6 | 0,64 | 0,14 |  |  | 49.94 (85.4) | 13.37 (13.41) |
| precentral.L | 14 | 0,37 | 0,13 | 0,72 |  |  | 71.72 (77.41) | 62.3 (63.81) |
| precentral.R | 14 | 1,4 | 0,62 | 0,17 |  |  | 46.87 (87.08) | 11.89 (15.38) |
| rostralmiddlefrontal.L | 14 | 1,5 | 0,57 | 0,15 |  |  | 128.19 (204.59) | 44.58 (52.06) |
| rostralmiddlefrontal.R | 14 | 1,5 | 0,63 | 0,15 |  |  | 70.07 (97.4) | 27.65 (27.48) |
| superiorfrontal.L | 14 | 1,4 | 0,54 | 0,18 |  |  | 98.15 (145.21) | 43.08 (28.84) |
| superiorfrontal.R | 14 | 1,8 | 0,67 | 0,096 |  |  | 90.76 (132.2) | 27.43 (29.42) |
| superiorparietal.L | 14 | 1,5 | 0,57 | 0,17 |  |  | 86.66 (118.29) | 36.49 (46.93) |
| superiorparietal.R | 14 | 1,5 | 0,61 | 0,15 |  |  | 61.4 (107.46) | 16.45 (22.89) |
| superiortemporal.L | 14 | 1,1 | 0,38 | 0,31 |  |  | 91.21 (114.81) | 54.38 (76.19) |
| superiortemporal.R | 14 | 1,3 | 0,53 | 0,22 |  |  | 75.11 (117.26) | 30.63 (38.45) |
| supramarginal.L | 14 | 1,7 | 0,53 | 0,11 |  |  | 109.34 (125.95) | 55.83 (44.1) |
| supramarginal.R | 14 | 1,6 | 0,65 | 0,12 |  |  | 67.67 (112.69) | 17.66 (18.47) |

**References**

1. Destrieux, C., et al., *Automatic parcellation of human cortical gyri and sulci using standard anatomical nomenclature.* Neuroimage, 2010. **53**(1): p. 1-15.

2. Tadel, F., et al., *Brainstorm: a user-friendly application for MEG/EEG analysis.* Comput Intell Neurosci, 2011. **2011**: p. 879716.
